# Postoperative Pain at Discharge From the Post-anesthesia Care Unit: A Case-Control Study

**DOI:** 10.7759/cureus.72297

**Published:** 2024-10-24

**Authors:** Ximena M Aladro Larenas, Maribel Castillo Cuadros, Irving E Miguel Aranda, Cristian I Ham Armenta, Horacio Olivares Mendoza, Mariana Freyre Alcántara, Irina Vázquez Villaseñor, Gabriel Villafuerte Jiménez

**Affiliations:** 1 Anesthesiology, Hospital Ángeles Lomas, Mexico City, MEX; 2 Research and Development, Actipulse Neuroscience, Cambridge, USA

**Keywords:** anesthesia, logistic regression model, machine learning, pain predictors, postoperative pain management

## Abstract

Introduction: Despite advancements in postoperative pain management, approximately 20% of patients still experience severe pain within the first 24 hours post-surgery. Previous studies utilizing machine learning have shown promise in predicting postoperative pain with various models. This study investigates postoperative pain predictors using a machine learning approach based on physiological indicators and demographic factors in a Mexican cohort.

Methods: We conducted a retrospective case-control study to assess pain determinants at Post-anesthesia Care Unit (PACU) discharge at Hospital Ángeles Lomas in Mexico City. Data were collected from 550 patients discharged from the PACU, including 292 cases and 258 controls, covering a range of surgical procedures and illnesses. Machine learning techniques were employed to develop a predictive model for postoperative pain. Physiological responses, such as blood pressure, heart rate, respiratory rate, and anesthesia type, were recorded prior to PACU admission.

Results: Significant differences were found between cases and controls, with factors such as sex, anesthesia type, and physiological responses influencing postoperative pain. Visual analog scale (VAS) scores at PACU admission were predictive of pain at discharge.

Conclusions: Our findings reinforce existing literature by highlighting sex-based disparities in pain experiences and the influence of anesthesia type on pain levels. The logistic regression model developed, incorporating physiological responses and sex, shows potential for refining pain management strategies. Limitations include the lack of detailed surgical data and psychological factors, and validation in a prospective cohort. Future research should focus on more comprehensive predictive models and longitudinal studies to further improve postoperative pain management.

## Introduction

Postoperative pain management is a critical aspect of patient care, affecting recovery trajectories, patient satisfaction, healthcare costs, and, more recently, opioid abuse prevention [1-3]. Despite advances in surgical techniques and anesthetic management [4,5], nearly 20% of patients continue to experience severe pain within the first 24 hours after surgery, a statistic that has remained almost unchanged over the past 30 years, increasing significantly their risk of developing chronic pain associated with the procedure [3,6].

The Post-anesthesia Care Unit (PACU) serves as the critical transition point between the operating room and hospital wards, where most patients spend the initial hours of their postoperative recovery. Effective pain management during this period is essential in reducing the risk of developing postoperative pain. Numerous studies have shed light on the factors contributing to poor acute postoperative pain management. A comprehensive systematic review, encompassing data from 48 studies and a substantial cohort of 23,037 patients, identified crucial predictors of postoperative pain such as preoperative pain, anxiety, age, and the nature of the surgical procedure [7]. Another meta-analysis, incorporating findings from 33 studies, underscored nine preoperative variables inversely linked to effective postoperative pain control. These included factors like young age, female sex, smoking, and a history of depressive symptoms or anxiety [8]. Furthermore, a meticulous review involving 14 studies delved into the correlation between preoperative responses to experimental pain stimuli and subsequent clinical postoperative pain experiences. Remarkably, this analysis revealed that preoperative pain testing had the capacity to predict between 4% and 54% of the variability in postoperative pain outcomes [9].

In addition to the description of the phenomenon, various methodologies have been employed to predict poor acute postoperative pain control using machine learning. For instance, a comprehensive study utilized five distinct algorithms, analyzing preoperative data from an extensive retrospective cohort comprising 8,071 surgical patients. The aim was to assess the risk of encountering moderate to severe postoperative pain [10]. In another investigation, the Perioperative Quality Improvement Program served as the foundation for developing and validating a logistic regression model. This model sought to predict severe pain on the initial postoperative day, drawing from preoperative variables and involving a substantial cohort of 17,079 patients undergoing major surgery. Notably, the model exhibited an enhancement in performance by incorporating perioperative covariates, highlighting the insufficiency of relying solely on preoperative variables for accurate postoperative pain prediction [11]. Furthermore, a study focused on machine learning applications in a dataset featuring 13,700 ambulatory surgery patients was performed to anticipate opioid requirements at two distinct post-surgical stages. Among the tested models, the Random Forest model emerged as superior, boasting a prediction accuracy of 72%. This outcome highlighted the critical influence of procedure type, patient medical history, and procedure duration as pivotal factors in forecasting opioid needs [12].

Our study aims to enrich the existing literature through a case-control design, with a specific focus on postoperative pain experienced at the point of discharge from the PACU in a Mexican healthcare setting. Previous studies and data sets, mainly based on different cultural and geographic landscapes, may not be directly applicable to the Mexican population due to cultural variations in pain perception and management [13,14]. This study addresses that gap by developing a machine-learning model tailored to the Mexican healthcare environment, leveraging population-specific characteristics. Our goal is to assess the predictive power of known postoperative pain variables, such as physiological responses and demographic factors, within this context. Additionally, we aim to identify any unique predictors that may be specific to this population, thereby enhancing our understanding of postoperative pain prediction and contributing to more personalized pain management strategies in Mexican healthcare.

## Materials and methods

Study design

We conducted an unmatched case-control study at Hospital Ángeles Lomas, a tertiary-level healthcare facility in the metropolitan area of México City. Our study harnessed data retrieved from the hospital's digital health record system and all data were anonymized to ensure that researchers could not identify individual patients. Ethical approval was provided by the ethics committee of Hospital Ángeles Lomas under registration number 480/2023.

Setting

The study incorporated patients admitted to the PACU of the healthcare facility over a six-month timeframe, spanning from November 15, 2022, to April 01, 2023.

Participants

The eligibility criteria for cases encompassed patients who reported pain scores equal to or greater than 2 on a visual analog scale (VAS) upon discharge from the PACU. In contrast, controls reported VAS scores of either 1 or 0. Exclusion criteria comprised all pediatric subjects (those under 18 years old). Furthermore, only records that included all essential variables were considered for inclusion in the study.

Variables

The study systematically examined an extensive array of variables to offer a comprehensive understanding of factors influencing postoperative pain. These variables encompassed demographic characteristics such as age and sex, duration of time spent in the PACU, and various clinical measurements recorded both upon entry to the PACU and at the point of discharge. These clinical metrics included Ramsay Scale scores, heart rate, respiratory rate, arterial pressure, and oxygen saturation levels. Additionally, the analysis explored relevant clinical parameters such as the American Society of Anesthesiologists (ASA) Physical Status Classification System, the type of anesthesia administered during surgery, the utilization of antagonists (sugammadex), the method of airway support, and the administration of analgesia during the PACU stay. The analgesia administered included morphine, buprenorphine, tramadol, or ketorolac, depending on the severity of the patient’s pain. 

Bias

The study is susceptible to potential biases, with selection bias being a notable concern since only records with complete variables were included. This approach might inadvertently exclude specific patient demographics or clinical presentations, introducing a potential limitation to the study's generalizability. Moreover, the exclusive focus on adults and reliance on electronic health records, which may inherently contain inaccuracies, could introduce additional biases. These factors should be considered when interpreting the findings and extrapolating them to broader populations or clinical contexts.

Statistical analysis

The statistical analyses and model development were conducted using Python 3.7 (Python Software Foundation, Wilmington, DE) to explore the dataset characteristics. Initial assessments of normality were performed through histograms and the Shapiro-Wilk test (data not shown). Due to the non-normal distribution of the data, medians (x͂) and interquartile ranges (IQR) were reported for continuous variables, while frequencies and percentages were employed for categorical data.

To assess the differences in continuous variables between case and control groups, a Mann-Whitney U-test was employed. Categorical variables were compared using a chi-square test. A significance level of p <0.05 was considered indicative of statistical significance. Odds ratios (OR), along with their corresponding 95% confidence intervals (CI), were calculated for categorical variables using contingency tables.

During the predictive modeling phase, we first conducted feature selection based on pre-PACU availability and a p-value threshold of 0.1 from the case-control group comparison. Features that met these criteria were then used to train a logistic regression model. The model was implemented using the liblinear solver, with L2 regularization (ridge) applied to prevent overfitting, setting the regularization strength parameter C to 1.0. The optimization process was configured to run for a maximum of 1000 iterations, with a tolerance level of 1x10^-4^ to ensure convergence.

Before training, we performed data preprocessing steps that included imputation and standardization of numerical features, as well as imputation and one-hot encoding of categorical features. The dataset was then stratified by the target variable and split into training (80%) and testing (20%) sets to maintain representative distribution across subsets. The logistic regression model was trained on the training set, and its performance was evaluated on the test set using metrics such as accuracy, sensitivity, specificity, F1-score, and area under the receiver operating characteristic curve (AUROC).

The source code for the predictive model is available at https://github.com/neuro-gabo/cureus-postoperativepain-casecontrol.

## Results

**Figure 1:**
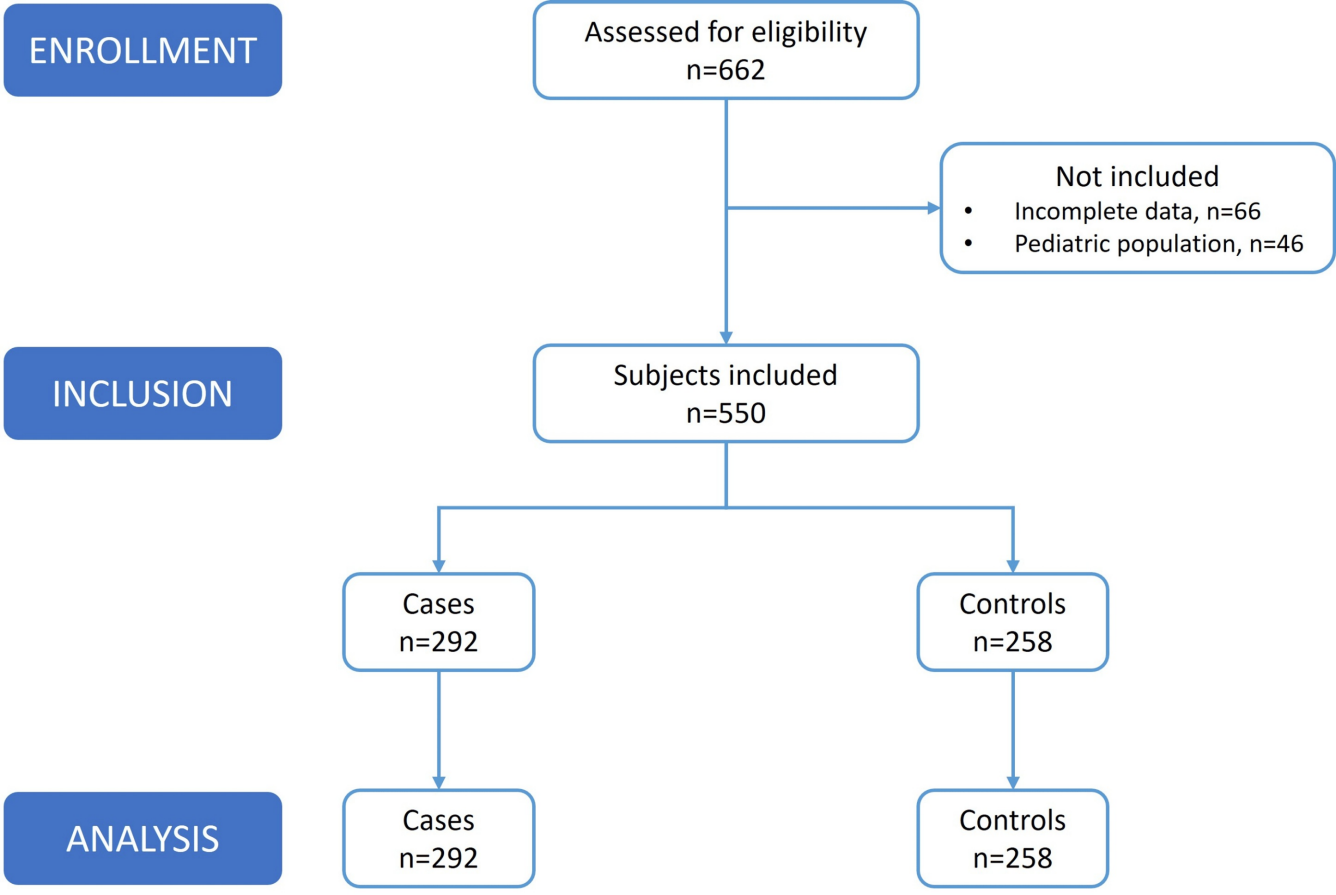
Participants’ selection process and inclusion criteria throughout the study.

During the specified period, 662 subjects underwent surgery and were admitted to the PACU. Of these, 550 met the inclusion criteria, and the final analysis comprised 292 cases and 258 controls (Figure 1). The median VAS score at discharge for the case group was x͂=3 (IQR=2-4), while for the control group, it was x͂ =0 (IQR=0-0).

**Figure 2:**
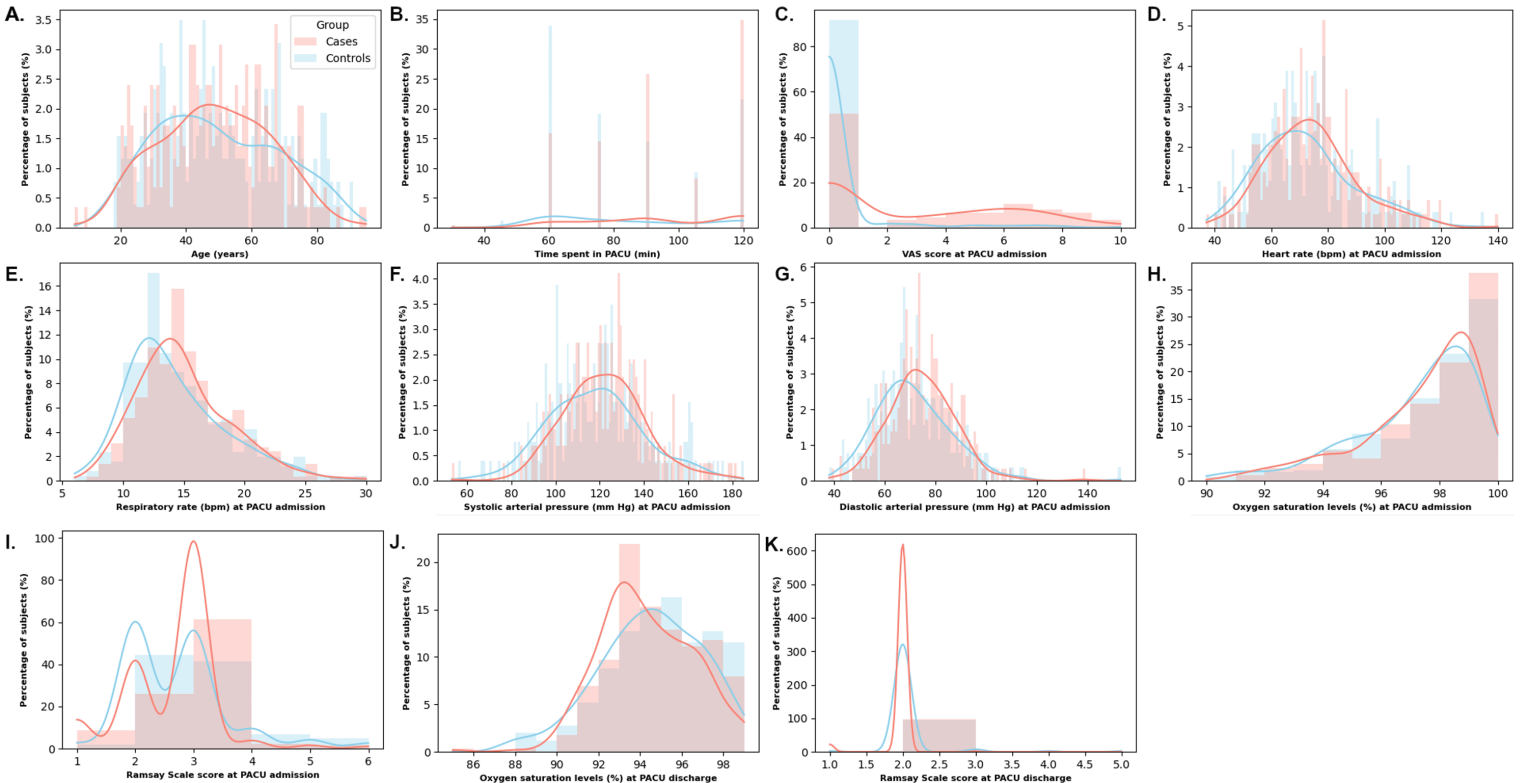
Continuous variables examined within both the case (red, n=292) and control (blue, n=258) groups. Distribution of continuous variables within the subjects: age (a), time spent in the PACU (b), VAS scores at PACU admission (c), heart rate (beats per minute) at PACU admission (d), respiratory rate (breaths per minute) at PACU admission (e), systolic arterial pressure (mm Hg) at PACU admission (f), diastolic arterial pressure (mm Hg) at PACU admission (g), oxygen saturation levels (%) at PACU admission (h), Ramsay Scale scores at PACU admission (i), oxygen saturation levels at PACU discharge (j), and Ramsay Scale scores at PACU discharge (k). PACU, Post-anesthesia Care Unit; VAS, visual analog scale.

The statistical analysis revealed significant differences between cases and controls across various parameters. Table 1 presents data from continuous variables, while Figure 2 provides a visual representation. Notably, the median time spent in the PACU was significantly longer for cases (x͂=90 min, IQR=75-120) compared to controls (x͂=75 min, IQR=60-105), with a p-value of less than 0.0001.

**Table 1:** Continuous variables examined within both the case and control groups. Significant differences were assessed using Mann-Whitney U-tests (p<0.05, n=550). PACU, Post-anesthesia Care Unit; VAS, visual analog scale; IQR, interquartile range, SatO_2_, oxygen saturation level.

	Variable	Median (IQR)		p-Value
		Controls (n=258)	Cases (n=292)	
	Age (years)	48 (34.25-65)	49 (36-62)	0.7738
	Time spent in the PACU (min)	75 (60-105)	90 (75-120)	<0.0001
At PACU admission	VAS score	0.00 (0.00-0.00)	0.00 (0.00-6.00)	<0.0001
	Heart rate (beats per minute)	70.00 (60.00-82.00)	73.00 (63.00-82.00)	0.1008
	Respiratory rate (breaths per minute)	13.00 (12.00-16.00)	14.00 (12.00-17.00)	0.0101
	Systolic arterial pressure (mm Hg)	119.00 (102.25-131.00)	122.00 (110.00-133.25)	0.0144
	Diastolic arterial pressure (mm Hg)	69.50 (61.00-80.00)	73.00 (66.75-82.25)	0.0010
	SatO_2 _(%)	98.00 (96.00-99.00)	98.00 (96.00-99.00)	0.3120
	Ramsay Scale score	3.00 (2.00-3.00)	3.00 (2.00-3.00)	0.5090
At PACU discharge	SatO_2 _(%)	95 (93-96)	94 (93-96)	0.1577
	Ramsay Scale score	2.00 (2.00-2.00)	2.00 (2.00-2.00)	0.0004

Upon examining variables recorded at PACU admission, the distribution of VAS scores differed markedly between groups (x͂=0, IQR=0-0 for controls vs. x͂=0, IQR=0-6 for cases, p<0.0001). Furthermore, median systolic and diastolic blood pressures were lower in controls compared to cases (p=0.0144 and p=0.001, respectively), while respiratory rates were notably higher in the case group (p=0.0101). No significant differences between groups were observed regarding age, heart rate, oxygen saturation levels, or Ramsay Scale scores at PACU admission. However, at PACU discharge, VAS scores exhibited significant disparities between controls and cases (p<0.0001), as did Ramsay Scale scores (p=0.0004), whereas oxygen saturation levels did not differ between groups.

Table 2 presents data pertaining to categorical variables. Notably, a higher proportion of female subjects was found in the case group (71.92%) compared to the control group (63.57%), with this difference proving statistical significance with a p-value of 0.0451. The frequency of receiving analgesia during the PACU stay exhibited a significant difference between the groups, with 74.66% in cases and 20.16% in controls (p<0.0001). Concerning the type of anesthesia administered during surgery, distinct utilization rates were noted for balanced general anesthesia, with 66.78% in cases versus 54.65% in controls (p=0.0047), and regional anesthesia, accounting for 3.77% in cases compared to 10.47% in controls (p=0.0035).

**Table 2:** Categorical variables examined in the case and control groups. Significant differences were compared using chi-square tests (p<0.05, n=550). ASA, American Society of Anesthesiologists; PACU, Post-anesthesia Care Unit; NIV, non-invasive ventilation; OR, odds ratios; CI, confidence intervals.

Variable		Controls (n=258)		Cases (n=292)		OR (95% CI)	p-Value
		Count	%	Count	%		
Sex	Female	164	63.57	210	71.92	1.4679 (1.0243, 2.1035)	0.0451
	Male	94	36.43	82	28.08	0.6813 (0.4754, 0.9762)	
ASA score	ASA I	74	28.68	97	33.22	0.8716 (0.6197, 1.2259)	0.4820
	ASA II	157	60.85	168	57.53	1.2369 (0.8600, 1.7790)	0.2915
	ASA III	26	10.08	26	8.9	0.8722 (0.4925, 1.5444)	0.7464
	ASA IV	1	0.39	1	0.34	0.8832 (0.0550, 14.1921)	1.0000
Type of anesthesia used during surgery	Balanced general	141	54.65	195	66.78	1.6681 (1.1808, 2.3566)	0.0047
	Balanced general + Regional	7	2.71	4	1.37	1.9417 (0.7790, 4.8397)	0.2188
	Balanced general + Sedation	1	0.39	5	1.7	0.8832 (0.0550, 14.1921)	1
	Balanced general + General intravenous	1	0.39	1	0.34	0.8832 (0.0550, 14.1921)	1
	Regional	27	10.47	11	3.77	0.3349 (0.1626, 0.6897)	0.0035
	Sedation	9	3.49	10	3.42	0.3843 (0.1169, 1.2630)	0.1767
Use of antagonists	No	178	68.99	194	66.44	0.8897 (0.6216, 1.2735)	0.5840
	Yes	80	31.01	98	33.56	1.1240 (0.7853, 1.6088)	
Type of airway support during PACU stay	Facial tent	144	55.81	205	70.21	1.8654 (1.3131, 2.6501)	0.0007
	Nasal cannula	97	37.6	74	25.34	0.5634 (0.3912, 0.8114)	0.0026
	Reservoir mask	12	4.65	12	4.11	0.8786 (0.3876, 1.9914)	0.9194
	Thermal humidifier	3	1.16	1	0.34	0.2921 (0.0302, 2.8258)	0.5306
	High-flow nasal oxygen (NIV)	2	0.78	0	0	NA	NA
Use of analgesia during PACU stay	No	206	79.84	74	25.34	0.0857 (0.0573, 0.1282)	<0.0001
	Yes	52	20.16	218	74.66	11.6705 (7.8029, 17.4551)	

Furthermore, the prevalence of facial tent usage during the PACU stay was significantly higher among cases (70.21%) compared to controls (55.81%, p=0.0007). Conversely, nasal cannulas were more commonly employed in controls (37.6%) than in cases (25.4%, p=0.0026).

No significant differences were observed between controls and cases for other combinations of anesthesia, nor for the use of reservoir mask, thermal humidifier, or high-flow nasal oxygen (non-invasive ventilation); ASA scores and the use of antagonists did not exhibit significant differences between the groups.

**Figure 3:**
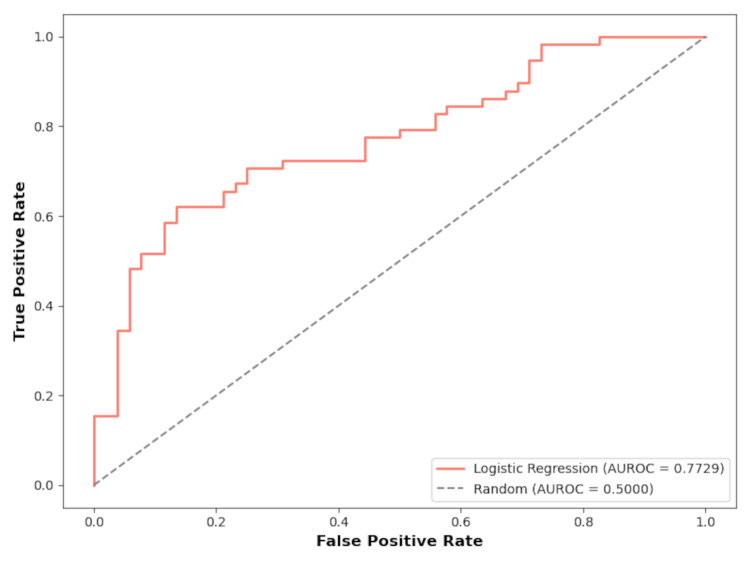
Depiction of the performance of the logistic regression model through a ROC curve. The AUROC value, measured at 0.7729, signifies the model's discriminative ability. A value of 0.7729 suggests that the model demonstrates good discrimination in distinguishing between the presence or absence of postoperative pain. ROC, receiver operating characteristic; AUROC, area under the ROC curve.

The logistic regression model included the variables VAS score at PACU admission, systolic and diastolic blood pressure, respiratory rate, type of anesthesia, and patient sex. Categorical variables were one-hot encoded to ensure proper integration into the model. The performance of the logistic regression model on the test set underwent thorough evaluation using multiple metrics. The model demonstrated an accuracy of 0.72, signifying the proportion of correct predictions. In detecting pain at PACU discharge, the sensitivity and specificity were 0.75 and 0.71, respectively, yielding an F1-score of 0.73. For predicting the absence of pain at PACU discharge, the sensitivity and specificity were 0.69 and 0.73, respectively, resulting in an F1-score of 0.71. The model's discriminatory power, as measured by the AUROC score, was 0.7729 (Figure 3).

## Discussion

Our study aimed to explore various factors that contribute to pain levels at PACU discharge, focusing on a cohort from the Mexican healthcare system. We identified that prolonged PACU stays, sex-based differences, and the type of anesthesia administered are significant predictors of pain at discharge. Specifically, women experienced higher levels of pain, and patients receiving balanced general anesthesia reported more intense pain compared to those given regional anesthesia. Additionally, our findings suggest that the type of respiratory support used, such as facial tents or nasal cannulas, may also influence pain levels. Understanding the interplay between these predictors can enhance the development of tailored pain management strategies. 

The association between prolonged stays in PACU and increased pain upon discharge is supported by previous research [15,16]. A study identified a correlation between initial pain levels upon PACU admission and the duration of PACU stay [17]. Specifically, patients experiencing severe pain upon admission tended to require longer stays. However, due to the retrospective nature of our dataset, the precise relationship between our outcome variable and PACU stay duration is not fully clear. It is possible that an extended PACU stay could signify additional undocumented complications, potentially influencing pain levels at discharge.

In our study, the higher prevalence of women in the case group experiencing postoperative complications is consistent with existing literature on sex-based disparities in postoperative pain. Numerous studies have shown that women often report higher levels of postoperative pain compared to men, particularly following thoracic, cardiac, orthopedic, and neurosurgical procedures [18-22]. However, not all studies report such disparities; some find no significant difference in postoperative pain between sexes [23,24]. This inconsistency may be influenced by confounding factors such as age, type of surgery, and study design. A key limitation of our study is the lack of sex matching in our cohorts, which complicates the interpretation of our findings. This highlights the need for further research that addresses sex-specific factors in pain management. A deeper understanding of these differences is essential for developing more targeted interventions for postoperative pain management [21].

In line with previous research, our findings indicate that the type of anesthesia significantly influences postoperative pain outcomes. Specifically, we found that patients administered balanced general anesthesia exhibited heightened pain levels upon PACU discharge, compared to those receiving regional anesthesia. This aligns closely with Cabedo et al.'s findings, whose study demonstrated that regional block techniques, used alone or in conjunction with general anesthesia, were linked to lower pain levels compared to sole general anesthesia [25]. Other studies have found similar data, with patients under general anesthesia reporting higher postoperative pain levels [26,27]. A key mechanism behind this observation could be the prevention of central sensitization by regional anesthesia. Central sensitization, a phenomenon where the central nervous system undergoes a process of increased sensitivity and hyperexcitability to pain stimuli, plays a pivotal role in the development of chronic and severe postoperative pain. The use of regional anesthesia may mitigate this process, thereby reducing both the intensity and the potential chronicity of postoperative pain [25,28].

We observed a significant correlation between the type of respiratory support device used in the PACU and the prevalence of pain at discharge. Patients using facial tents reported higher levels of pain, whereas those using nasal cannulas experienced less pain at discharge. To our knowledge, this observation has not been previously reported in the literature. This finding suggests that the type of respiratory support may serve as a predictive variable for pain outcomes. Specifically, facial tents are often utilized for patients with more severe preoperative complications or those undergoing complex surgical procedures, which may contribute to increased pain levels at discharge. In contrast, nasal cannulas are generally preferred for patients with fewer complications or less complex surgeries, indicating smoother surgical outcomes and correspondingly lower pain levels at PACU discharge. Thus, while the use of facial tents might indicate more serious conditions or challenging surgeries, it is important to recognize that this may also reflect the patients' pre-existing health status and surgical complexity. Future research should explore the interplay between respiratory support methods, patient characteristics, and pain outcomes to better understand this relationship.

The VAS score at PACU admission was a significant predictor of pain at PACU discharge, highlighting the direct relationship between initial pain levels and subsequent pain experiences, while the concurrent presence of altered respiratory rate and arterial pressure at PACU admission reflects the patient’s pain experience. Therefore, early pain levels serve as critical indicators of potential pain outcomes at discharge.

The logistic regression model we developed for predicting pain at PACU discharge demonstrates the growing importance of predictive models in the field of anesthesia. By incorporating variables relevant to the Mexican population, such as physiological responses and demographic factors, our model attempts to address potential cultural and healthcare-specific differences in pain management. Our model, which included the variables VAS score at PACU admission, systolic and diastolic arterial pressure, respiratory rate, type of anesthesia, and patient sex, showed promising results with an accuracy of 0.72 and an AUROC of 0.7729. Other similar models that incorporate a larger set of variables, including social history and psychiatric or neurological diagnoses, had attained similar accuracy and AUROC [11,12]. In a clinical setting, this model could be a valuable tool for anesthesiologists and postoperative care teams. Predicting the likelihood of pain at PACU discharge can guide clinicians in tailoring pain management strategies more effectively from the onset of recovery. For example, higher predicted pain scores could prompt early interventions, such as adjustments in analgesic administration or closer monitoring of vital signs.

Our study has certain limitations. A significant constraint is the lack of detailed surgical data, including the types of surgeries performed and specific outcomes of the surgery (blood loss, associated complications, etc.). Research indicates that different types of surgeries and surgical outcomes can cause varying types and amounts of pain, influencing postoperative pain management and recovery [7,8,29]​​. Furthermore, our dataset did not include psychological factors, which are critical in the perception and management of pain. Psychological states like anxiety and depression have been shown to have a profound impact on postoperative pain, sometimes comparable to the influence that the physical aspects of surgery have on postoperative pain [7,8,30].

Regarding the predictive model, while logistic regression is a widely used method, its limitations in handling complex, non-linear relationships in clinical data can reduce its predictive accuracy and generalizability. This is especially pertinent in medical data, where interactions between variables can be intricate and not necessarily linear. Future studies might benefit from considering other predictive models that can better capture these complexities. Additionally, while we have calibrated our model using retrospective data, validating the model prospectively in a real-time clinical setting will be crucial to assess its predictive accuracy and ensure its robustness across different patient cohorts. 

Finally, our study's focus on pain at the time of discharge from the PACU, without subsequent follow-up, restricts our understanding of the progression and duration of postoperative pain beyond this period. This limitation underlines the importance of longitudinal studies in future research to track pain levels over time, providing a more comprehensive picture of postoperative pain management and recovery.

## Conclusions

Our study highlights the key factors influencing postoperative pain at PACU discharge in a Mexican cohort, such as anesthesia type, respiratory support, and sex-based differences. The predictive model we developed shows potential, but its applicability is limited by the retrospective nature of our data and the exclusion of variables like psychological factors and detailed surgical data. Future research should focus on refining this model and validating it in real-time clinical settings. This approach could lead to more precise pain management strategies, improving patient outcomes and optimizing resource allocation within the Mexican healthcare system.
